# The Mitochondrial m.3243A>G Mutation on the Dish, Lessons from In Vitro Models

**DOI:** 10.3390/ijms241713478

**Published:** 2023-08-30

**Authors:** Sanna Ryytty, Riikka H. Hämäläinen

**Affiliations:** A.I. Virtanen Institute for Molecular Sciences, University of Eastern Finland, Neulaniementie 2, 70211 Kuopio, Finland; sannary@uef.fi

**Keywords:** m.3243A>G, mitochondria, mtDNA, heteroplasmy, cell model, cybrid cell, iPSC

## Abstract

The m.3243A>G mutation in the tRNA Leu(UUR) gene (MT-TL1) is one of the most common pathogenic point mutations in human mtDNA. Patient symptoms vary widely and the severity of the disease ranges from asymptomatic to lethal. The reason for the high heterogeneity of m.3243A>G-associated disease is still unknown, and the treatment options are limited, with only supportive interventions available. Furthermore, the heteroplasmic nature of the m.3243A>G mutation and lack of specific animal models of mtDNA mutations have challenged the study of m.3243A>G, and, besides patient data, only cell models have been available for studies. The most commonly used cell models are patient derived, such as fibroblasts and induced pluripotent stem cell (iPSC)-derived models, and cybrid models where the mutant DNA is transferred to an acceptor cell. Studies on cell models have revealed cell-type-specific effects of the m.3243A>G mutation and that the tolerance for this mutation varies between cell types and between patients. In this review, we summarize the literature on the effects of m.3243A>G in cell models.

## 1. Mitochondria

Mitochondria are ubiquitous intracellular organelles that originate from the endosymbiosis of a proteobacterial ancestor, but, over time, they have developed into an essential part of mammalian cells [1]. Their key function is energy production, and they generate about 90% of cellular adenosine triphosphate (ATP) through the oxidative phosphorylation (OXPHOS) process in most mammalian cells. Each nucleated human cell typically contains several hundreds of mitochondria, depending on the tissue’s energy needs. In addition to energy production, mitochondria also play important roles in a series of signal pathways and various metabolic processes, including the tricarboxylic acid cycle (TCA), β-oxidation of fatty acids, calcium homeostasis, cell cycle regulation, and regulating cell death through apoptosis.

Mitochondria are surrounded by a double membrane structure which is formed of lipid and protein-rich inner and outer membranes [2]. The outer membrane is smooth and contains various porin proteins, allowing for the diffusion of small molecules across the membrane. The inner membrane, on the other hand, is impermeable to most solutes and folded into cristae structures that enlarge the surface area. As a result of its selective permeability, an electrochemical gradient is generated across the inner membrane. The inner membrane accommodates the energy-generating complexes of the electron transport chain (ETC), which generate ATP via OXPHOS, a process in which electrons are transferred via complexes I-IV to generate a proton gradient that drives ATP synthesis via complex V. The electrochemical membrane potential across the inner membrane is essential for mitochondrial function and ATP production.

Roughly 1500 proteins are needed for the proper structure and function of mitochondria [1]. The majority of these proteins, 99%, are encoded by nuclear genes, synthesized in cytoplasm, and imported into mitochondria. However, mitochondria also possess their own genetic material, mitochondrial DNA (mtDNA). This is a circular, double-stranded DNA molecule with 16,569 base pairs in humans and it encodes 13 respiratory chain proteins essential for oxidative phosphorylation. In addition, mtDNA encodes the 22 tRNAs and 2 rRNAs required for mitochondrial protein translation. Each mitochondrion contains multiple copies of mtDNA, which can either all be identical, i.e., homoplasmic, or one mitochondria can encompass two or multiple types of mitochondrial genomes in a heteroplasmic state. Similar to the nuclear genome, mtDNA can also harbor pathological mutations. The mutation rate of mtDNA is 10–100 times higher than that of nuclear DNA (nDNA), with ~6 × 10^−8^ point mutations per base pair generated yearly [3]. Most human disease-causing mtDNA mutations are heteroplasmic and a variety of both point mutations and deletions in mtDNA are known to cause human disease [4].

Mitochondria are highly dynamic and continuously undergo fusion and fission, responding to the energy needs of a cell [1]. Redundant or dysfunctional mitochondria can be eliminated from cells via mitophagy, the mitochondrial specialized form of autophagy. However, rescuing a cell from dysfunctional mitochondria is not always possible and the dysfunction can lead to mitochondrial disease.

## 2. Mitochondrial Disease

Mitochondrial diseases are a clinically and genetically heterogeneous group of metabolic disorders characterized by dysfunction in oxidative phosphorylation. They are among the most common human metabolic diseases, with a prevalence of around 23.3 per 100,000 [5]. They can be caused by mtDNA or nDNA mutations; these mutations can be inherited or sporadic and the age of onset, as well as the range and severity of symptoms, show considerable variations between patients [6]. While the clinical symptoms between mitochondrial disease patients may vary significantly, tissues with a high energy demand, such as neuronal and muscular tissues, are usually the most affected ones. There is no cure for mitochondrial disease and, thus far, treatments mainly strive to relieve symptoms and slow down the progression of the disease.

Most pathogenic mutations in mtDNA are heteroplasmic and the biochemical phenotype only manifests functionally when the level of the mutated mtDNA reaches a critical threshold [7]. The threshold for biochemical deficiency is mutation and cell-type dependent and may even vary for the same mutation in different patients. However, a pathogenic mutation is normally required to reach a relatively high level (>70%) before a deleterious biochemical phenotype manifests at a single-cell level. The clinical phenotype can also vary markedly between patients carrying the same mtDNA mutation at apparently similar heteroplasmy levels. One example of this is the m.3243A>G disease, where a marked phenotypic variability between patients is likely to reflect the nuclear genetic factors influencing the expression of the disease [8].

## 3. m.3243A>G Mutation

The m.3243A>G mutation in the tRNA Leu (UUR) gene (MT-TL1) is one of the most common pathogenic point mutations in human mtDNA [5,9]. The prevalence of the mutation varies between populations. For example, in the Finnish population, it is reported to be 16.2 per 100,000 [10], while a high frequency of 236 per 100,000 is seen in Australia [9]. Mitochondrial disease associated with the m.3243A>G mutation is typically a clinically heterogeneous neurogenetic disorder [11]. However, the phenotype seen in m.3243A>G patients is highly complex, presenting a great variety in manifestation and severity ranging from asymptomatic to lethal disease. The clinical phenotype can involve various organs, such as the brain, skeletal muscles, heart, endocrine system, gastrointestinal tract, eye, ear, and skin. Some patients conform to well-established syndromes, while in some, symptoms appear individually or are associated with other syndromes, making clinical diagnosis difficult [12]. The m.3243A>G-associated disease is often progressive and increases the risk of sudden death and a reduced lifespan. However, both the progression of symptoms and influence on patients’ life expectancy are difficult to predict.

The m.3243A>G mutation was first identified in mitochondrial encephalopathy, lactic acidosis, and stroke-like episodes (MELAS) syndrome patients in 1990 [13]; hence, the m.3243A>G mutation is often referred to as the MELAS mutation. MELAS, together with maternally inherited diabetes and deafness (MIDD) syndrome, represent the two most frequent clinical outcomes of the m.3243A>G mutation [12,14]. In total, 80% of all MELAS cases present m.3243A>G, but under 20% of all m.3243A>G carriers manifest MELAS, whereas MIDD is the most common clinical outcome in m.3243A>G patients [11,14,15,16].

m.3243A>G is mostly seen in a heteroplasmic state, but the exact thresholds for its different pathologies are not fully defined. A variation in the level of m.3243A>G heteroplasmy in the brain has been associated with phenotypic heterogeneity in MELAS [17]. However, even though a high mutation load has been seen to correlate with disease burden, this correlation is not always direct and drastic variation between individuals is evident. High mutation levels in specific patient tissues do not automatically lead to a specific phenotype and nuclear genetic factors are very likely to influence the clinical outcomes of m.3234A>G patients [8]. Furthermore, the heteroplasmy levels can vary dramatically between the tissues of a patient. Post-mitotic tissues, such as muscle and neural tissues, tend to have higher and more stable levels of heteroplasmy than the more accessible mitotic tissues, such as blood, urinary epithelial cells, and buccal mucosa, complicating molecular genetic diagnosis in many patients [16]. The greatest decrease in mutation load is seen in blood, where the levels of m.3243A>G fall by an estimated 1.4–2.3% per year [18,19]. However, when age-correlated, the heteroplasmy level in blood correlates relatively well with the disease burden and progression in patients [19,20]. Some studies have suggested that the m.3243A>G mutation amount could correlate with the clinical phenotype and that a high mutation load leads to MELAS syndrome [13], while lower mutation levels result in MIDD syndrome [21]. However, these correlations are not fully clear, and the individual variation between patients is large. Furthermore, over a 75% m.3243A>G mutation load in the central nervous system of a MIDD patient without the MELAS phenotype has been reported, suggesting that high mutation levels do not automatically lead to a specific phenotype [22]. The role of the mutation load in m.3243A>G disease needs to be further investigated, but research is complicated by a lack of relevant tissue samples from patients.

## 4. Molecular and Functional Consequences of the m.3243A>G Mutation

**Figure 1:**
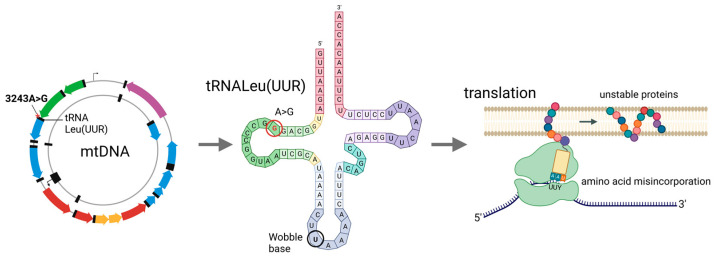
m.3243A>G mutation. A schematic showing the location of the m.3243A>G mutation in mtDNA and its effect on the tRNALeu(UUR) and translation. Image created by BioRender.com, accessed on 16 August 2023.

The m.3243A>G variant affects the MT-TL1 gene (Figure 1), which encodes the mitochondrial tRNA Leu(UUR), a 75 bp long tRNA for leucine [11]. The transition of adenine into guanine in the position 3243 affects the D-loop of the tRNALeu(UUR) molecule, and induces alterations in structure, stability, and codon recognition.

The m.3243A>G mutant tRNA-Leu(UUR) lacks post-transcriptional taurine modifications at the wobble U base (Figure 1), and specifically reduces UUG translation, with no specific effect seen on UUA translation, resulting in the misincorporation of amino acids to all the mitochondrial proteins encoded by mtDNA (Figure 1) [23]. The unstable protein products, in turn, lead to electron transport chain defects. Proteins rich in UUG codons, such as the ND6 subunit of complex I, are mainly affected by the m.3243A>G mutation [24], and while all mitochondrially encoded respiratory chain subunits can be affected, the m.3243A>G mutation primarily seems to result in a deficiency of complex I [11]. In addition to taurine modifications, tRNA conformational changes [25], a lack of 3′-end processing, CCA addition [26], aminoacylation [23], and transcription termination defects [27] have been suggested to underlie translational defects. In addition to the tRNA-related effects, it has been suggested that the m.3243A>G mutation may also affect ribosomal RNAs, including impairing 16S rRNA transcription termination [27].

## 5. Cell Models of m.3243A>G

Genetic manipulation of the mitochondrial genome has turned out to be much more difficult than the manipulation of the nuclear genome. The mitochondrial double membrane is not easily penetrated by polynucleotides, which makes the introduction of RNA or DNA into the mitochondria extremely difficult. This, together with the multicopy nature of the mitochondrial genome, has led to a lack of efficient methodology for mtDNA modification. This has long prevented the generation of animal models for specific mitochondrial mutations. The newly developed, purely protein-based base-editing methodology has now opened up possibilities for creating animal models of mtDNA mutations [28,29]; however, cell models still dominate the studies of m.3243A>G effects. When reviewing the cell studies of any mtDNA mutation, two major factors are important to account for. Namely, the specific cell type in question and the mutation load. The mutation load naturally affects the manifestation and severity of the defect. In many cases, heteroplasmic mtDNA mutations need to exceed a specific threshold for a pathogenic state to manifest [30]. Furthermore, for some mutations, different mutation loads lead to different symptoms, and the higher the mutation load, the more severe the symptoms may be. For m.3243A>G, the specific thresholds for its various symptoms have not been determined reliably. Furthermore, the mutation loads can vary significantly between the different tissues of each patient. Several studies have tried to systematically analyze the response of cells to the mutation level [31,32,33]. These studies, surprisingly, have suggested that the response of a cell to an increasing mutation level is not always linear. For example, a 60–70% m.3243A>G mutation load has been shown to increase the mtDNA protein-coding gene expression in comparison to both higher and lower mutation loads [31,32].

In addition to the mutation amount, the cell and tissue type are also important. While similar outcomes can be seen in various cell types, the effect of the m.3243A>G mutation can vary dramatically between different cell types [34,35,36]. For example, different respiratory chain (RC) defects can be seen in different cell types of the same patient [34], dysfunctional mitochondria are more evident in A549.B2 than RD.Myo cybrids, and the mutations modulate the mitochondrial dynamics and mitophagy in a cell-type-dependent manner in cybrid cells [35]. Furthermore, the effect of the mutation can also vary between different patients, even in the same cell type [37,38]. For example, Matsubara and co-authors reported that the same mutation load affects the mitochondrial respiration of iPSCs from one patient, whereas it has no effect in the cells of another patient [37], and we have shown that, in iPSC-derived cardiomyocytes, m.3243A>G decreases the ATP levels in the cells of a cardiomyopathy patient, but not in the cells of another patient without cardiac symptoms [38]. These findings suggest that additional nuclear factors are likely to affect the outcome of the m.3243A>G mutation.

Due to the tissue- and cell-type specific effects, it is important to study the effect of the m.3243A>G mutation in various cell types. Highly metabolic tissues, which commonly are the most severely affected, such as the brain and cardiac tissue, are not easily accessible in live patients and heteroplasmy assessments from autopsy samples may not be available or may be influenced by tissue necrosis, leading to unreliable results in some cases. In the following chapters, we will review the published data on m.3243A>G in vitro models, with a focus on the mutation levels and cell-type specificity of the phenotype. Both novel stem-cell-derived models and more traditional cybrid models will be discussed.

### 5.1. Fibroblast Studies

Fibroblasts isolated from dermal skin biopsies are the most used models when patient-derived cells are needed. They are relatively easily available from patients, easy to culture, and proliferate well for several passages in vitro, which allows for the expansion and cryopreservation of the cell material. Dermal manifestations are seen in some primary mitochondrial diseases, many skin disorders have mitochondrial components, and mitochondria are the primary organelle affected in aging skin, supporting the importance of mitochondria for fibroblast homeostasis [39]. The effects of the m.3243A>G mutation have also been successfully studied in fibroblasts. Generally, the mutation levels in patients’ fibroblast cultures are fairly modest, however, subclones with varying mutation loads, including high mutation levels, can be derived by cloning, but the options can be limited in specific patients. However, Yokota and coworkers showed that it is possible to obtain fibroblast lines with up to a 100% m.3243A>G mutation amount using the cloning approach [36]. In patient fibroblast studies, control cells are generally derived from healthy volunteers, making it difficult to isolate the mutation effect from additional biological variations between individuals.

Even though fibroblasts are not highly oxidative cells, clear effects of the m.3243A>G mutation can be seen in them, and surprisingly low mutation levels have been reported to induce functional effects in fibroblasts. The proliferation of fibroblasts is affected in patient cultures, where already a 30% mutation load has been reported to reduce the growth rate of fibroblasts [40], and the growth ability is severely decreased in fibroblasts with a high mutation frequency (>95%) [36]. m.3243A>G mutant fibroblasts are also highly glucose dependent, and culturing in a low glucose concentration further decreases their proliferation rate [40]. Glucose uptake, glycolytic metabolism, and lactate production are increased in m.3243A>G fibroblasts and, once a 50% mutation threshold is reached, the increasing mutation level is reported to correlate with the cellular lactate levels [36], suggesting increasing glycolytic activity as the mutation load increases. In contrast, the m.3243A>G mutation decreases both the level and activity of the respiratory chain complexes in fibroblasts [41,42]. Specifically decreased Complex I (CI) and Complex IV (CIV) activities have been widely reported [36,40,41,42,43]. In line with this, decreased oxygen consumption has been seen in m.3243A>G fibroblasts, and the defect is more severe in cells with a high (86%) mutation load than in cells with lower (30%) mutation levels [40]. Defects in energy metabolism are reflected in the ATP levels in fibroblasts and already a low (17–43%) mutation load has been reported to decrease the ATP levels when compared to control cells [41,42]. However, ATP levels do not seem to correlate with mutation load and no significant differences in ATP levels are seen in cells with mutation levels between 50% and 100% [36].

Some studies have reported functional effects in fibroblasts already at extremely low mutation levels, suggesting that fibroblast cultures could be sensitive to even very low m.3243A>G mutation loads. Mutation levels under 10% (4–9%) were reported to decrease mitochondrial membrane potential and increase mitophagy in fibroblasts in one study [42]. The membrane potential has also been shown to be affected in other studies, and a high (86%) mutation level decreases the membrane potential more than a moderate 30% mutation level [40]. Alterations in mitochondrial mass, biogenesis, and turnover have also been reported, with low mutation levels (4–9%) being reported to increase mitochondrial biogenesis proteins and mitochondrial mass, whereas a higher mutation load seems to decrease mitochondrial biogenesis [42]. Furthermore, an increased level of autophagy markers has been seen in m.3243A>G mutant fibroblasts [41,42], but this seems to be due to the accumulation of autophagosomes due to their decreased elimination rather than increased mitophagy and mitochondrial turnover [41].

While the m.3243A>G mutation tends to generally decrease mitochondrial function, increased mtDNA copy numbers have been detected in mutant fibroblasts [33]. This effect seems to be patient dependent and may indicate a compensatory response to the presence of pathogenic mutations in certain patients.

Interestingly, adenosine monophosphate-activated protein (AMPK) activation has been detected in fibroblasts with mutation levels under 10%, while a similar response was not evident in cells with higher mutation levels [40,42]. The pharmacological activation of AMPK signaling in fibroblasts with higher mutation loads decreased reactive oxygen species (ROS) levels, increased ATP levels, and normalized autophagy. These results indicate that fibroblasts with low mutation levels may protect themselves against RC deficiency and increasing ROS levels via AMPK activation. Why the same protective mechanism does not apply to cells with higher mutation levels is not known. Cotan and colleagues detected decreased coenzyme Q (CoQ) levels in mutant fibroblasts (40–70% mutation level) and CoQ supplementation in fibroblasts has shown similar protective effects as AMPK activation [40,41,42]. CoQ is a cofactor in the electron transport chain and shows both antioxidant functions and energetic effects.

Besides AMPK signaling, the PI3K-Akt-mTORC1 pathway, the important regulator of cell proliferation, is also associated with the m.3243A>G defect in fibroblasts. Chung and coworkers showed that m.3243A>G promotes changes in the metabolites associated with the upregulation of the PI3K-Akt-mTORC1 axis and induces the chronic activation of this axis in fibroblasts [40]. Furthermore, they showed that the pharmacological inhibition of PI3K-Akt-mTORC1 signaling improves the mitochondrial function and decreases the glucose dependence in m.3243A>G mutant fibroblasts, and interestingly, also reduces the mutation load, suggesting an effect on mitochondrial biogenesis and/or turnover.

A wide range of different m.3243A>G mutation levels, from very low at 4% [42] to over 90% mutation loads [36,43], has been reported in fibroblast studies, and fibroblasts indeed seem very sensitive to the effects of the m.3243A>G mutation. An already 4% mutation load has been shown to affect fibroblasts, and drastic effects have been reported when this mutation level reaches 17% [42]. However, as none of these studies have used isogenic controls, some of the reported effects may not be solely due to mtDNA mutations and differences between patients are evident. A summary of the effects seen in fibroblasts, as well as the cell types derived from them, is presented in Table 1.

### 5.2. Stem Cell Based Models

#### 5.2.1. Pluripotent Stem Cells

Induced pluripotent stem cells (iPSC) are a valuable model for the study of mitochondrial DNA mutations. iPSCs can be generated from basically any adult cell type, with the most used starting cell types being dermal fibroblasts and peripheral blood cells, these being the cells most commonly available from patients and requiring the least invasive methods for sample collection. As the m.3243A>G mutation load tends to decrease significantly in patient blood, the common starting material in these patients is usually dermal fibroblasts. In many m.3243A>G patients, the mutation load in their fibroblasts also tends to be on the lower side. However, while Yokota and coworkers suggested that reprogramming does not significantly change the mutation load between iPSCs and fibroblasts [36], several other studies have reported large variations in the mutation levels between different iPSC clones derived from the same fibroblasts [33,34,45,46]. This segregation of mtDNA mutations is considered to arise due to the mitochondrial genetic bottleneck, a phenomenon described first in germ cells and later also during embryogenesis [47,48,49]. This bottleneck effect enables the derivation of isogenic iPSCs lines with high mutation loads, together with lines with extremely low and even non-detectable mutation levels from the same fibroblasts.

During the past 10 years, several studies utilizing iPSC lines from m.3243A>G patients have been published. Most studies have not detected any defects in the reprogramming process itself due to these mutations. However, fibroblasts with a very high mutation load, at over 90%, showed an impaired reprogramming efficiency in one study [36]. While some mtDNA mutations seem to be selected against in stem cells [50], this does not seem to be true for the m.3243A>G mutation. However, due to the mitochondrial bottleneck effect, the arising iPSC clones may have very different mutation levels than the original starting cells and a biphasic segregation of the mutations during reprogramming, tend to shift the mutation levels towards high or low rather than moderate mutation levels [33,34]. Altered mtDNA copy number levels, both in the starting fibroblasts as well as during the reprogramming process, could be a potential indicator for some compensatory response to the pathogenic m.3243A>G mtDNA mutation, in turn aiding with cellular reprogramming.

After reprogramming, the heteroplasmy levels have been shown to be quite stable in established iPSCs [44]. However, both decreasing mutation levels during the first month of culture and increasing mutation levels during long-term culture have been reported [44,46]. Furthermore, clone-dependent differences in the stability of these mutation levels have been reported [36,46], indicating that it is essential to verify the mutation levels regularly in long-term cultures. A putative mechanism underlying the different behavior of the mutations in stem cells could arise from patient- and clone-specific differences in mitochondrial biogenesis or mtDNA replication and turnover.

While reprogramming fibroblasts with heteroplasmic m.3243A>G mutations allows for the generation of isogenic iPSC clones with variable mutation amounts, the quality control of these clones is important. As for any iPSC clone, karyotyping the lines is necessary to verify that the cells do not harbor any chromosomal abnormalities. In addition to nDNA, mtDNA can also harbor additional changes between clones [46]. A next-generation sequencing analysis of the mtDNA in iPSC clones, with and without m.3243A>G mutations, revealed that heteroplasmic mtDNA mutations present at low levels in parental fibroblastsmay become enriched in iPSC clones up to the levels where they show functional consequences. Thus, the sequencing of mtDNA has been proposed as a selection criterion for all iPSC clones.

The effect of the m.3243A>G mutation on iPSC function has been studied in detail. iPSCs are relatively glycolytic cells without vast oxidative needs and thrive in hypoxic conditions. It is probably due to this that m.3243A>G mutations have not been seen to exert major effects on iPSCs and, in comparison to fibroblasts, iPSCs seem to tolerate well even high mutation levels [36]. Further, while the functional effects seen in iPSC studies have been milder, the mutation levels have typically been significantly higher in iPSC studies than those in fibroblast studies. Similar to fibroblasts, a CI defect has been reported in m.3243A>AG mutant iPSCs, but the threshold for this defect is high and has varied from over 70% to over 80% in different studies [37,44]. Further, instead of a specific CI defect, a compensatory upregulation of CII levels was detected in cells with high mutation levels in one study [34]. No other RC defects have been reported in published iPSC studies. Despite the differences in mutation thresholds, all these studies have suggested that iPSCs tolerate relatively high m.3243A>G mutation levels.

While high mutation loads seem to result in a CI deficiency in iPSCs, the effect on the function of the respiratory chain is not as straightforward and seems to be patient-dependent [37]. Most studies have reported no respiration defects, even in cells with high (70 to 90%) mutation levels [33,51]. However, Matsubara and colleagues studied cells from two independent patients and detected reduced mitochondrial oxygen consumption in the iPSCs derived from one of the patients, whereas a similar defect was not seen in the other patients’ cells. However, they did not detect any effect on the cellular ATP levels in the iPSCs of either patient, suggesting that, even with decreased respiratory function, iPSCs can still fulfill their energy demand through other compensatory channels [37].

**Figure 2:**
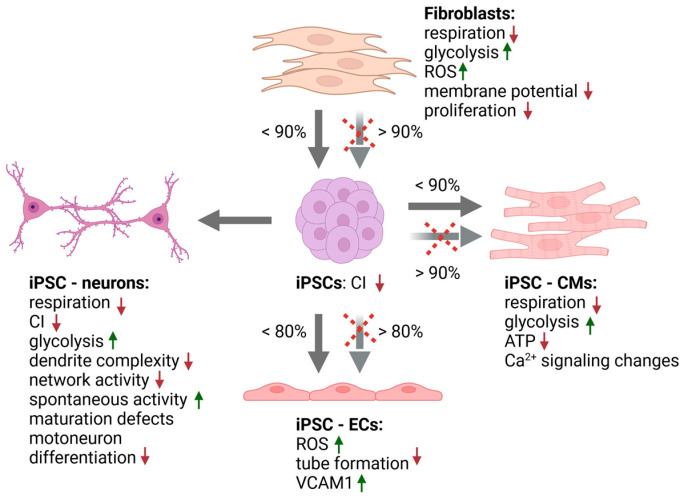
A summary of the effects of m.3243A>G in fibroblasts, induced pluripotent stem cells (iPSCs), and iPSC-derived cells. High mutation load reduces reprogramming efficiency as well as differentiation of iPSCs (Arrows crossed with red). Cell-type-specific effects are listed under the cell type (EC = endothelial cells, CM = cardiomyocytes, red arrow down = decrease, and green arrow up = increase.) Image created by BioRender.com, accessed on 16 August 2023.

IPSCs enable studies on patient-derived cell types that are typically not available from patients but are commonly affected in m.3243A>G patients, such as neurons and cardiomyocytes, which have been widely studied. Studies on iPSC-derived endothelial cells and retinal pigment epithelial cells have also been published [52,53]. In the majority of these studies, mutant iPSCs have shown a normal differentiation ability to specific tissue or cell types, and iPSCs with high m.3243A>G mutation levels have been shown to spontaneously differentiate towards all three germ layers [34,44]. This agrees with the normal early development of patients with symptoms developing only later in life. Furthermore, even homoplasmic m.3243A>G mutations have been shown to not affect the pluripotency of iPSCs [36]. However, a few studies have suggested that very high mutation levels could affect cardiac and endothelial differentiation [51,52]. In the following sections, we will summarize the findings from iPSC-derived cell models (Table 1, Figure 2).

Overall, even high m.3243A>G mutation loads do not drastically affect the energy levels or mitochondrial membrane potential in iPSCs, and the majority of studies have shown the normal proliferation and differentiation of mutant iPSCs towards different cell types, further supporting the view that m.3243A>G does not significantly affect either the mitochondrial function or stem cell properties of iPSCs

#### 5.2.2. Neural Cells

Neurological defects are the most common symptoms in m.3243A>G-related disease, and iPSCs offer vast opportunities for studying the relation between the mutation and neural cell function. Neural progenitor cells (NPCs) are tissue-specific progenitor cells of the central nervous system (CNS) that differentiate into the glial and neuronal cell types that populate the CNS. In in vitro studies, NPCs are an important intermediate stage when differentiating neuronal cells from iPSCs, and a number of studies have reported the normal formation of m.3243A>G mutant NPCs from iPSCs [34,51,54]. However, in one study, a very high 90% mutation load was reported to impair neuronal differentiation, leading to collapsed neurospheres and defects in NPC formation [51]. On the other hand, defects in the maturation of neuronal cells have been seen in several studies. While m.3243A>G mutant cells (71% mutation load) were able to differentiate into excitatory cortical neurons, delayed maturation and increased cell death were detected in one study [45]. In another study, iPSCs with an 80% mutation load were able to generate spinal organoids, but compared to isogenic control cells, they showed neurogenesis defects, including impaired neurite outgrowth and delayed neural maturation, which were associated with increased Notch signaling [54]. Interestingly, treatment with the gamma secretase inhibitor DAPT, which is known to inhibit Notch signaling [55], rescued the neurogenesis defects, suggesting that the hyperactive Notch indeed underlies these defects [54]. Notch signaling has been reported to positively influence mitochondrial fission, suggesting that proper mitochondrial dynamics are critical during neuronal development [56].

Some m.3243A>G patients present with muscle weakness or show other myopathic findings, and in accordance with this, an 80% m.3243A>G mutation load was reported to prevent the differentiation of motor neurons [54]. Taken together, these results suggest that, while the early stages of neuronal development are not significantly affected, functional and maturation defects are seen in more specialized neuronal cell types and a very high m.3243A>G mutation load may even prohibit neuronal formation.

At the respiratory chain level, m.3243A>G has been seen to decrease the CI levels in neurospheres [34], but no effect on cellular respiration or ATP production has been reported in NPCs [51]. However, in more mature iPSC-neurons, m.3243A>G mutations decrease mitochondrial respiration and ATP production while increasing glycolysis [45]. Defects in neuronal function have been seen in cells with a >65% mutation load, including a reduced dendritic complexity, fewer excitatory synapses, reduced network activity, and asynchronous network bursting, while increased spontaneous activity has also been detected. However, a 30% mutation load did not induce functional defects in iPSC-neurons [45], suggesting that the threshold for neuronal symptoms is above 30%.

The molecular mechanism leading from the m.3243A>G mutation to neuronal defects is not clear. Neurons have a high requirement for energy, and thus defects in energy production affect their function; however, additional defects are likely also involved in the pathogenesis of mitochondrial disease, as pure energy defects cannot really explain the complexity of patient phenotypes. Winanto and coworkers reported that Notch signaling, which, for example, maintains the stem cell identity in NPCs, is elevated in mutant spinal NPCs, which could explain the neurogenesis defects detected in their study [54]. CI deficiency upregulates Notch effector genes and is also associated with the maturation of NPCs, further suggesting that Notch signaling could be one of the pathways through which mitochondrial defects affect neuronal cells.

Excitatory neurons derived from m.3243A>G mutant iPSCs showed reduced neuronal network activity and synchronicity and downregulated the genes related to synaptic processes and mitochondrial respiration [45,57]. Interestingly, treatment with a novel, trial-phase mitochondrial disease drug, sonlicromanol, reversed the changes in gene expression; however, the effect was patient specific and not beneficial in all patients’ cells [57]. Sonlicromanol acts as an antioxidant and redox modulator, thus suggesting that altered redox homeostasis or increased oxidative stress may underlie some of the neuronal defects.

Earlier this year, a study utilizing direct transdifferentiation of m.3243A>G mutant fibroblasts to neurons (iNs) was published [43]. The benefits of this model are that age-associated phenotypes can be better detected in these iNeurons, whereas, as the reprogramming process erases the epigenetic memory of cells, they are generally absent from iPSC-derived neurons. In iNeurons, a combined CI and CIV defect was detected in cells with high mutation loads, and accordingly, the mitochondrial dynamics and respiration were also disturbed in the cells with over a 68% mutation loads, whereas the cells with a 20% mutation load showed normal mitochondrial function. Further, the membrane potential was decreased and the mitochondrial ROS levels were increased only in the iNs with a very high mutation load of >95%. These results suggest a direct correlation between the mutation load and severity of the mitochondrial defects in iNs.

#### 5.2.3. Cardiac Cells

Cardiomyopathy is one of the clinical symptoms associated with the m.3243A>G mutation, and approximately 30–50% of patients develop cardiac symptoms [58,59,60]. iPSC-derived cardiomyocytes (iPSC-CMs) are a unique model for mitochondrial cardiomyopathy studies, as patients’ cardiac cells are seldom available for research purposes. A very high mutation load of >90% was seen to inhibit cardiac development in one study [51], but mutation levels under 90% have not been seen to disturb the differentiation of iPSCs to cardiomyocytes [38,46,51].

Moderate and high (60 to 90%) m.3243A>G mutation levels have been shown to decrease the mitochondrial respiration and increase the glycolysis in iPSC-CMs in comparison to isogenic control cells with negligible mutation levels [38,46]. Further, the respiration defect was comparable in cells with either a 40% or 90% mutation level [51], suggesting that the defect does not correlate linearly with the mutation load, but rather, once the threshold (40%) is reached, similarly manifests independently of the mutation amount.

Similar to what has been reported from neuronal cells, we detected patient-specific responses to m.3243A>G mutations also in iPSC-CMs [38]. While iPSC-CMs from a patient without cardiac symptoms were able to increase glucose uptake and maintain cellular ATP levels despite a high 80% m.3243A>G mutation load, similar and lower (60–80%) mutation levels in the cells of a patient suffering from severe cardiomyopathy led to an ATP deficiency and altered Ca-signaling, also suggesting an altered contractile function in this patient’s cells.

#### 5.2.4. Other iPSC- Derived Cell Types

Vascular involvement in MELAS neuropathological features has been proposed and there is evidence of atherosclerotic lesions in patients’ aortic tissues [61]. Thus, Pek and co-authors studied iPSC-derived endothelial cells (iPSC-ECs) from an m.3243A>G patient [52]. When compared to isogenic control cells, an 80% mutation load both reduced the efficiency of the iPSCs in differentiating into iPSC-ECs and affected the functionality of the generated iPSC-ECs. The mtDNA mutations increased mitochondrial biogenesis and inhibited vascular tube formation. Mutant cells further showed increased oxidative stress and an increased level of oxidized, low-density lipoproteins (oxLDLs). The expression of vascular cell adhesion protein 1 (VCAM1), a member of the immunoglobulin family mediating the adhesion of inflammatory cells to endothelia and suggested to play a role in atherosclerosis development, was also increased. In agreement with this, monocytes had a higher tendency to adhere to the mutant ECs than their isogenic controls, suggesting an atherosclerosis-like pathology. Antioxidant treatments decreased the mitochondrial ROS levels, enhanced the vascular tube formation, and reduced the number of adhering monocytes in the mutant iPSC-ECs, thus further pointing towards oxidative stress as the underlying cause for the defects in endothelial cells.

Pigmentary retinopathy is one of the phenotypes associated with the m.3243A>G mutation [62], and Bhattacharya and colleagues differentiated iPSCs with various m.3243A>G mutation loads into retinal pigment epithelial cells (iPSC-RPECs) [53]. No effect was seen on the differentiation efficiency, but a significant reduction in the mitochondrial respiration of the iPSC-RPECs was detected. At the same time, the mutations also increased the reliance on glycolysis for energy production. The mutation amount was correlated with reduced adenosine monophosphate-activated protein kinase α (AMPKα) activation and increased signal transducer and activator of transcription 3 (STAT3) activation, further implicating altered energy metabolism in the mutant cells. The mutations further showed a negative correlation with autophagic activity, which was associated with aberrant lysosomal function and the failure of mitochondrial recycling.

### 5.3. Cybrid Models

**Figure 3:**
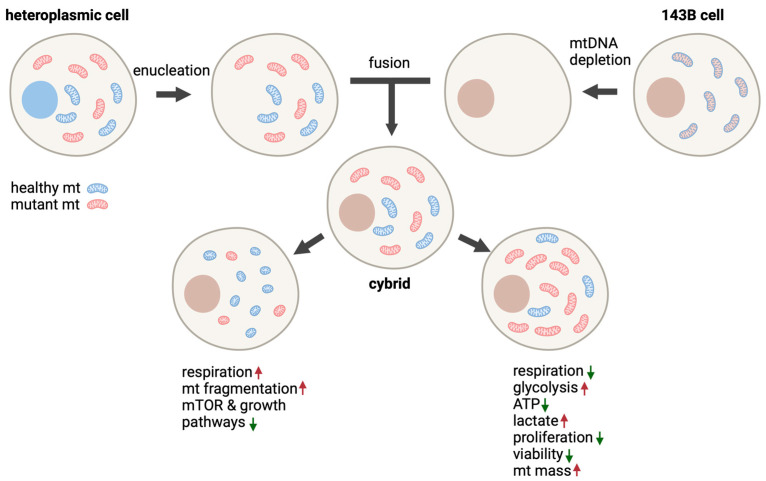
Production of cybrid cells and the effect of m.3243A>G in 143B cybrids. In cybrid cells, the nucleus of the cell is derived from an acceptor cell and mitochondria from donor cells. Mutation load between cybrid clones naturally varies, which enables selection of clones with specific mutation loads for the studies. Low (down left) and high (down right) m.3243A>G mutation load has been shown to affect 143B cybrids differently. mt = mitochondria, WT = wild type, arrow up = increase, and arrow down = decrease. Image created by BioRender.com, accessed 16 August 2023.

The most commonly used cell model for the m.3243A>G mutation has long been cybrid (cytoplasmic hybrid) cells [63]. Cybrid cells are hybrids of two cells, where one cell is nucleated and one is enucleated (Figure 3). The nucleated cell is a rho(0) cell, where the endogeneous mitochondria has been depleted. The benefit of cybrid cells is that mtDNA mutations can be studied in various nuclear backgrounds, or alternatively, different mtDNA haplotypes or mutations can be studied in the same nuclear background, and the mtDNA contribution to the phenotype can be isolated. Another benefit is that highly proliferative cells, such as carcinoma cells, can be utilized. However, as cancer cells adapt their metabolism to proliferation and biomass production though a Warburg effect, it is important to remember that their energy metabolism is quite different from that of most post-mitotic cell types [64]. To date, several different cybrid cell lines for the m.3243A>G mutation have been created. In this chapter, we review the results of m.3243A>G cybrid lines from 143B human osteosarcoma cells and SH-SY5Y human neuroblastoma cells. In addition, some specific aspects from other cybrid models, such as A549 human lung adenocarcinoma cells, RD.Myo human rhabdomyosarcoma cells, and U87MG glioblastoma cells, are discussed.

Studies with cybrid cell lines often utilize very high mutation loads of >90%. However, cybrid cells also permit studies with varying mutation loads and on the correlation between the heteroplasmy level and severity of symptoms. Studies on patient-derived cells have clearly shown that there are patient-specific differences in responses to the primary mtDNA mutation, indicating that the nuclear background may play a significant role in disease development. In most m.3243A>G cybrid studies, the focus has been on comparing mutant and healthy mtDNA, and not much consideration has been given to nuclear background, even though cybrid technology could be used to study the effect of different nuclear genomes on the expression of the mtDNA disease.

#### 5.3.1. 143B Human Osteosarcoma Cells

143B cybrid cells with the m.3243A>G mutation were published at the beginning of the 1990s [65,66]. They are the most used cybrid cell model for this mutation and large amounts of data about the mutation mechanism, as well as the effect of the mutation load, have been generated with 143B cybrids. The results obtained from these osteosarcoma cells have further been extended to explain the disease mechanisms in other tissue types, such as the role of m.3243A>G in pancreatic cells and diabetes [67,68] and neuronal and cardiac pathology [69,70].

Many published studies use 143B cybrids with very high m.3243A>G mutation loads of 90–100%. These studies have shown that a high m.3243A>G mutation load decreases cell proliferation [71] and viability [72,73] and increases the expression of mtDNA-encoded transcripts, together with increased mitochondrial mass [71] in 143B cybrids. However, while the mitochondrial mass is increased, these mutations decrease the amount of respiratory chain complexes [31,71,72], oxygen consumption [68,72,73], NAD+/NADH ratio [32], and ATP levels [42,69]. In agreement with a reduced respiration rate, 143B cybrids with high m.3243A>G mutation levels are highly glucose dependent, and in the absence of glucose, their ATP production is very modest [67]. However, while mutations increase the glucose utilization in 143B cybrids, they also decrease glucose oxidation, revealing an ineffective use of glucose for energy production. In addition, while the m.3243A>G mutation does not drastically affect the ROS levels in 143B cybrids [67,69], in the absence of glucose, the ROS levels are increased in mutant cybrids when compared to control cells [67]. Furthermore, the oxidation of fatty acids is decreased [68] and lactate production and glycolysis are increased in 143B cybrids with a high m.3243A>G mutation load [31,67,72], which supports the view that the mutant cells are unable to properly utilize oxygen and mitochondria for their energy production.

Although a high m.3243A>G level induces drastic metabolic changes in 143B cybrids, lower 20–30% mutation loads seem to have a very different effect. Interestingly, a 30% m.3243A>G mutation load has been seen to increase both oxygen consumption and glycolysis [68], however there was no significant effect on the expression of the genes related to glycolysis [31]. The low mutation load also stimulated glucose, pyruvate, and fatty acid catabolism [68]. 143B cybrids with 20–30% m.3243A>G mutation loads have also been shown to have reduced mtDNA-encoded tRNA levels, fragmented rounded mitochondria, a small cell size, and to downregulate growth-related pathways, such as mTOR signaling [31].

A transcriptomics analysis revealed a functional switch between 143B cybrids with 30% and 50% m.3243A>G mutation loads [31]. While a low level of mutations downregulates the growth pathways, higher mutation loads of over 50% upregulate the metabolic pathways related to both glycolysis and mitochondrial function and downregulate the genes involved in transmembrane signal transduction. The expression profiles associated with senescence and telomere maintenance have also been induced upon high mutation amounts.

Kopinski and colleagues studied the effect of the m.3243A>G mutation load on the nuclear epigenome through metabolic tracing in 143B cybrids and found that different mutation levels cause distinct metabolic and epigenomic changes [32]. Acetyl coenzyme A (acetyl-CoA) is the primary substrate for histone acetylation, while α-ketoglutarate (αKG) is a substrate for histone demethylation. Both are mitochondria derived. The study reported that a high m.3243A>G mutation load of 90–100% decreased the level of acetyl-CoA and H4K16 acetylation, whereas in cells with less than 70% mutant mtDNA, the acetylation levels were stable. In contrast, the αKG/succinate ratio was low in cells without mutations, increased in cells with intermediate mutation loads of 30–70%, and declined again when the majority of the mtDNA, >90%, was mutated, and the H3K9 di- and trimethylation correlated inversely with this result. The authors further reported that homoplasmic mutant cells showed decreased acetyl-CoA formation from glucose and increased the pyruvate metabolization to lactate, which limited the acetyl-CoA production. These results show that the metabolic changes caused by m.3243A>G mutations can affect the epigenetics of nDNA, possibly through altered pools of mitochondrial metabolites, and additionally, that the effect of m.3243A>G mutations on the nuclear epigenome does not correlate linearly with the mutation amount in 143B cybrids.

Together, the published studies on 143B cybrids show a clear response to different m.3243A>G heteroplasmy levels. A high >90% m.3243A>G level decreases the mitochondrial function and leads to proliferation and viability defects [71,72,73]. Intermediate m.3243A>G mutation loads of 50–90% result in enhanced mitochondrial function, as well enhanced glycolysis and transcriptional activity [31,32]. Low, 20–30%, mutation loads increase oxygen consumption, decrease cell size, and downregulate the growth pathways [31,68]. Additionally, the transcriptional profiles differ depending on the heteroplasmy levels. However, high and low m.3243A>G loads seem to induce quite similar changes in the genes of the metabolic pathways in 143B cybrids [68].

Transcriptomics studies have shown that m.3243A>G mutations induce multiple changes in the nuclear gene expression in 143B cybrids [31,68,70,74]. While the effect of m.3243A>G mutations on OXPHOS activity and mitochondrial energy production is straightforward, the impacts on other cellular pathways are more complex [31,68,74]. The changes in mitochondrial metabolites affect histone modification and can alter nDNA gene expression [32]. The nature of these changes, and whether they are protective or harmful, is mostly unknown.

Altered expressions of the genes of the TCA cycle and OXPHOS machinery, as well as changes in the genes linked to other energy metabolism pathways, such as fatty acid metabolism [68] and glycolysis [31], are commonly seen. Interestingly, many of these energy pathway changes seem to be specific for the m.3243A>G mutation, as similar changes in the TCA cycle or OXPHOS genes have not been detected in rho(0) 143B cells devoid of mitochondrial DNA [31,74]. Common changes within rho(0) cells are seen in ribosomal, RNA processing, peptide chain elongation, and protein translation pathways, suggesting that these alterations are general to all mitochondrial dysfunctions. Among the m.3243A>G-specific changes, pathways related to calcium and actin-binding activity, basement membrane, alanine and aspartate metabolism, TRAIL (Tumor necrosis factor-related apoptosis-inducing ligand)-induced apoptosis, and nucleotide excision repair are seen. Interestingly, several pathways related to neurodegenerative disorders are only affected in m.3243A>G 143B cybrids.

Putative therapeutic targets have been sought among the vast number of altered pathways identified in 143B cybrid studies. One candidate is the Mitochondrial Nuclear Retrograde Regulator 1 (MNRR1), which is required for the activation of mitochondrial unfolded protein response (UPRmt), a mitochondria-related stress response [75]. The MNRR1 levels are decreased in m.3243A>G mutant 143B cybrids. The overexpression of MNRR1 in these cells induces UPRmt and autophagy, enhances mitochondrial respiration and ATP levels, and decreases ROS levels. These results support a beneficial role for MNRR1 in m.3243A>G mutant 143B cybrids. Additionally, m.3243A>G mutant 143B cybrids have been seen to downregulate the genes involved in ubiquitin-mediated protein degradation, further supporting altered protein quality control as one of the m.3243A>G mechanisms [74].

A high m.3243A>G mutation load has also been shown to decrease the mRNA level of a novel mitophagy receptor, ATAD3B (ATPase Family AAA Domain Containing 3B), in 143B cybrids [76]. ATAD3B binds to LC3 and promotes oxidative-stress-induced mitophagy in a PINK1-independent manner, thus promoting the clearance of damaged mtDNA. Another interesting gene is mitochondrial leucyl-tRNA synthetase (LARS2). The carboxy-terminal domain of LARS2 (Cterm) interacts with the mutated mt-tRNAleu(UUR) gene and its precursor RNA19, with no effect on its steady-state levels or aminoacylation [72,73]. The LARS Cterm has been shown to increase the viability and oxygen consumption and decrease the apoptosis in 143B cybrids with m.3243A>G mutations, proposing possibilities for its use as a tool for rescuing cellular defects [73]. However, while Cterm seems to increase the viability of m.3243A>G mutant 143B cybrids, no effects on oxygen consumption, mitochondrial biogenesis, or mitophagy have been found [72]. Thus, the mechanism behind the increased cell viability is still unknown.

The effect of the m.3243A>G mutation on miRNA expression has also been studied in 143B cybrids. Homoplasmic m.3243A>G mutant cells significantly increase the ROS-related miRNA miR9/9*, which, in turn, downregulates the mitochondrial miRNA translation and modification-related genes GTP binding protein 3 (GTPBP3), Mitochondrial TRNA Translation Optimization 1 (MTO1), and tRNA mitochondrial 2-thiouridylase (TRMU) [77]. These effects are controlled by ROS via the NFkB pathway, lead to mt-tRNA hypomodification, and contribute to mitochondrial dysfunction in homoplasmic cybrids. Another study by the same authors identified 246 small RNAs with changed expressions between homoplasmic mutant and control cells, with 126 of the miRNAs being upregulated and 120 being downregulated [70]. These data show the upregulation of small RNAs as being related to TGF-β signaling and the epithelial to mesenchymal transition, as well as the downregulation of miRNA-regulated fetal cardiac genes. The study also identified significant changes in the expression pattern of mitochondrial tRNA fragments (mt tRFs), which may have a biologically relevant role in the pathology of mitochondrial diseases; however, further studies are needed to verify this and identify their functional role [78].

#### 5.3.2. SH-SY5Y Human Neuroblastoma Cybrids

The use of neural cell lines in in vitro studies is justified by the common occurrence of neuronal symptoms in m.3243A>G patients. SH-SY5Y is a thrice-subcloned neural cell line derived from the SK-N-SH neuroblastoma line. The cells can be differentiated to various types of functional neurons by specific growth factors and thus they serve as a good model for neuronal disorders. SH-SY5Y cells are also used as a m.3243A>G cybrid model.

In SH-SY5Y cybrids, a high, close to homoplasmy m.3243A>G mutation level decreases the CI and CIV activity [51,79,80,81]. However, the effects of more moderate mutation loads on RC complex activities are unclear. A 70% m.3243A>G mutation load has been reported both to decrease [79] and increase [80] CI activity. The reported drop in CI activity in these studies was, however, less drastic that that seen in cells with higher mutation loads. A high m.3243A>G mutation load also decreased the respiration [79] and increased the fragmentation of the mitochondria in SH-SY5Y cybrids [80]. However, no effect on cellular ATP levels has been seen in SH-SY5Y cybrids, probably due to the high glycolytic rate of these cells. m.3243A>G mutations increase the LDH activity, glucose consumption, alanine levels [80], and NADH/NAD ratio [81] in SH-SY5Y cybrids. Interestingly, when SH-SY5Y cybrids with a close to 100% m.3243A>G mutation load were cultured in low glucose conditions, their mutation load decreased to 90% and the RC complex activities were restored [80], suggesting that forced changes in metabolism can induce heteroplasmy shifting in these cells.

Glutamate is an important neurotransmitter and intracellular glutamate levels have been reported to correlate with m.3243A>G mutation loads, with an increasing mutation load also increasing the intracellular glutamate level [79]. However, while extracellular glutamate also increases in mutant SH-SY5Y cultures, its levels do not correlate with the mutation load, and homoplasmic mutant cultures have lower extracellular glutamate levels than cultures with moderate mutation loads, suggesting impaired glutamate transport in mutant cells. In some studies, m.3243A>G mutations have induced neuronal maturation defects and even neuronal cell death in SH-SY5Y cybrids [51]. A nearly homoplasmic, 98% m.3243A>G mutation level led to a 4-Aminobutyrate aminotransferase (ABAT) deficiency, the protein responsible for the catabolism of gamma-aminobutyric acid (GABA), the most important inhibitory neurotransmitter in the CNS [79]. Interestingly, ABAT also plays a role in mitochondrial nucleoside salvage and defects in it lead to mtDNA depletion [82]. A transcriptomics analysis revealed various changes in the nuclear gene expression in m.3243A>G mutant SH-SY5Y cybrids [79]. A total of 2046 genes were differentially expressed, and the main gene clusters were identified in glutamate and glutamine metabolism, GABA synthesis, release, reuptake, and degradation, and the TCA cycle.

SH-SY5Y cybrids have also been utilized in treatment trials. Ketone bodies (KBs) are a ketone group containing molecules produced from fatty acids. They are converted into acetyl-Coenzyme A, which is the substrate of the TCA cycle. KBs have been shown to exert beneficial effects in SH-SY5Y cybrids with a high (98%) m.3243A>G mutation load. KBs increased the mtDNA copy number, however, the mutation load was unaltered [81]. They also shifted the energy metabolism from glycolysis towards fatty acid oxidation and enhanced the mitochondrial respiration, CI activity, and mitochondrial networks, and decreased NADH/NAD+ ratio [79,81]. KBs were further shown to normalize the glutamate concentration in cells, suggesting that KBs could provide a treatment option for neural symptoms in m.3243A>G patients.

#### 5.3.3. Other Cybrid Models of m.3243A>G

Multiple additional cybrid models for the m.3243A>G mutation have been published. In this chapter, we discuss a few interesting findings from additional cell types.

Malena and colleagues compared the effect of m.3243A>G on two different cell types, human lung adenocarcinoma (A549) cybrids and human rhabdomyosarcoma (RD.Myo) cells [35]. Interestingly, the RD.myo cells seemed to favor the mutant over the wt mtDNA, while the A549 cells favored the wild-type mtDNA. Homoplasmic mutations decreased the membrane potential and ATP synthesis rate in both cell types, but decreased ATP levels were detected only in the A549 cybrids. These changes were not seen in either cell type with a more moderate mutation load. Instead, a moderate, 70–80% mutation load altered the mitochondrial dynamics in a cell-type-specific manner. The mutations increased the mitochondrial fragmentation, mtDNA turnover, and mitophagy in the A549 cybrids and these changes were correlated with the mutation loads. On the other hand, in the RD.Myo cybrids, the mitophagy markers were decreased and mitochondrial networks were elongated. These findings indicate that m.3243A>G mutations can modulate mitochondrial dynamics and mitophagy in a cell-type-dependent manner, even with opposite effects being seen in different cell types.

The underlying mechanism in MELAS stroke episodes is unknown. One option could be defects in the brain microvessels and blood–brain barrier (BBB). Davidson and coworkers thus studied the effect of m.3243A>G on the BBB in a unique model with immortalized astrocyte cybrids and endothelial cybrid cells with a high, 97% mutation load [83]. They found that m.3243A>G decreased the CI and CIV activity in both astrocytes and endothelial cells. Furthermore, m.3243A>G decreased the transendothelial electrical resistance (TEER) of the endothelial cells, suggesting an increased permeability and compromised integrity of the barrier, and the authors concluded that m.3243A>G can perturb BBB function.

## 6. Conclusions

Mitochondrial m.3243A>G mutations lead to variable symptoms in patients, and they can affect several different organs. The heterogeneity of clinical symptoms, together with the heteroplasmic nature of the m.3243A>G mutation, make mechanistic studies, diagnostics, and the treatment of patients challenging. To date, the effect of m.3243A>G has not been studied in animal models, signifying the importance of cell models for these studies. Even with the obvious limitations of in vitro cell models, they have greatly enhanced the understanding of the mechanisms behind the m.3243A>G disease. While new transgenic methodology enables the modification of mtDNA and opens up new avenues for m.3243A>G studies in animal models, the patient-specific defects seen in many cell studies warrant studies with human and patient cells in the future.

Patient studies have revealed the tissue specificity of m.3243A>G effects, which has been well replicated in cell studies where different effects have been reported in different cell types. For example, whole iPSCs tolerate m.3243A>G mutations relatively well, fibroblasts seem to be much more sensitive [36,42]. iPSC-derived cell models have, in recent years, shown effects on cell-type-specific functions, such as the electrical activity of neuronal cells [45] and calcium signaling of cardiac cells [38], that are likely important in determining the clinical outcome of patients. Studies using cybrid cell models initially revealed the different effects of mutations in different cell types. For example, a high m.3243A>G mutation load decreased the cellular energy levels in A549 cybrids, but not in RD.Myo cybrids, and opposite effects on mitochondrial dynamics were reported in these two cell types [35]. Various published studies have reported functional differences between the cells of different patients, replicating the variability seen in the clinic and signifying the importance of additional modifying factors for the disease outcome. However, the underlying mechanisms of this variability between different patients are still unclear [37,38] and warrant additional research, as understanding them could help clinicians to better target patients with treatments suitable for them. For example, sonlicromanol treatment was beneficial for the iPSC-neurons of certain patients, while cells from other patients did not benefit from the treatment [57].

The heteroplasmic nature of the m.3243A>G mutation complicates studies on several levels. Different mutation loads have been reported to exert different effects on cell models and these effects do not always correspond linearly to the mutation level. Typically, it is thought that, in diseases related to heteroplasmic mtDNA mutation, there is a threshold that must be achieved before symptoms appear. Furthermore, it has been suggested that the outcome of the disease would be dependent on the mutation level, so that, for example, a higher mutation load would trigger MELAS and a lower load MIDD. However, the threshold for the pathogenicity of the m.3243A>G mutation is far from clear. Cell studies have clearly shown that the tolerance for m.3243A>G depends on the cell type. This is also seen in patients, where tissues with a high energy demand are usually the most critically affected with symptoms. In the published cell studies, a very high mutation load of >90% is normally seen to have the most drastic effect on cells. However, such high mutation levels are seldom seen in patients, who may still manifest a very severe life-threatening disease [11]. Thus, studies performed on cells with lower mutation loads are very interesting. Studies with more moderate mutation levels have also shown much more variability between different tissues and different patients. For some tissues, the mutation load seems to correlate with the severity of the functional defect, while in other tissues, once a threshold is reached, the defect always manifests similarly. Further, different, sometimes even opposite, effects have been seen in different cell types with similar mutation levels. It would be interesting to see more studies on cells with moderate mutation loads, to further understand the relation between the mutations and the functionality of different cell types.

While biological variation between different patients, arising for example from age, biological sex, haplogroup background, and nuclear background, is likely to account for the heterogeneity of the published effects of the m.3243A>G mutation, additional aspects may also be involved. The majority of the published studies are performed on bulk cells, and little is known about the intercellular heterogeneity in heteroplasmy levels, which will certainly also be reflected in the functionality of the cells.

Numerous studies on various cell types with m.3243A>G mutations have been published and they have enhanced our understanding of the biology underlying the disease seen in patients. However, there is still much to do before we can fully understand the effects of m.3243A>G mutations in patients.

## Figures and Tables

**Table 1:** Summary of the effects of the m.3243A>G mutation reported in different cell types. n.d, not determined.

Cell Type/Phenotype	Fibroblasts	iPSCs	Neurons	Cardiac Cells
**Metabolism**	Decreased respiration. Increased glycolytic activity [40].	Decreased respiration in some patients [33,37,44].	Decreased respiration in some studies. Increased glycolytic activity [45].	Decreased respiration. Increased glycolytic activity [38,46].
**Mitochondrial effects**	Decreased membrane potential [40,41]. CI and CIV deficiency [35,40,41,42,43].	Membrane potential not affected. CI deficiency [33,44].	CI deficiency [34]	Membrane potential not affected. CI deficiency [38].
**Cellular energy**	Decreased ATP [40,41].	Not affected [37].	n.d	Decreased ATP [38].
**Functional defects**	Reduced growth rate [40].	n.d	Increased spontaneous activity, decreased synchronous activity, decreased excitatory synapses, and dendritic complexity [45].	Changes in calcium signaling [38].

## Data Availability

Not applicable.
